# Supplementary material for: Maternal hybrid immunity and risk of infant COVID-19 hospitalizations: national case-control study in Israel
Source: Nat Commun. 2024 Apr 2;15:2846. doi: 10.1038/s41467-024-46694-x (PMC10987618; doi:10.1038/s41467-024-46694-x)

Maternal hybrid immunity and risk of infant COVID-19 hospitalizations:

national case-control study in Israel

Supplementary Material

**Supplemental Table 1- Main medical diagnoses of hospitalized infants with positive SARS-CoV-2 test that were not included in the study outcome**	
**Main medical diagnosis:**	***n***
*Infectious disease	62
Covid-19 positive test at birth	21
Malformations and surgical problems	15
Metabolic problems	3
Acute events: seizures, arrythmia, dyspnea	4
Others	23
**Total**	**128**

* Infectious diseases include bacterial pneumonia, cellulitis, colitis, croup, meningitis, otitis media, mononucleosis, urinary tract infection, pyelonephritis, sepsis, and bronchiolitis. Viral diseases comprise herpes, rhinovirus, and RSV.

Supplemental Table 2: Effectiveness of Maternal Immunization Status Against Infant COVID-19-Related Hospitalization During the First 180 Days of Life, Among all Infants in the Cohort (before exclusions).

**Subgroup**	**Hazard Ratio (95% confidence interval) (Study Cohort)**	**Hazard Ratio (95% confidence interval) (Including non-eligible cases)**
**Naïve**	Reference group	Reference group
**Hybrid**	0.16 (0.10-0.25)	0.24 (0.17-0.37)
**Natural Immunity**	0.44 (0.32-0.61)	0.57 (0.44-0.73)
**Full vaccination (3 doses)**	0.34 (0.26-0.44)	0.37 (0.29-0.46)
**Partial vaccination (2 doses)**	0.71 (0.59-0.85)	0.72 (0.61-0.84

Supplementary Figure 1- Age Distribution of Infant cases.

X-axis- Infant age at hospitalization (in days) y- axis- The number of cases hospitalized on a given day.

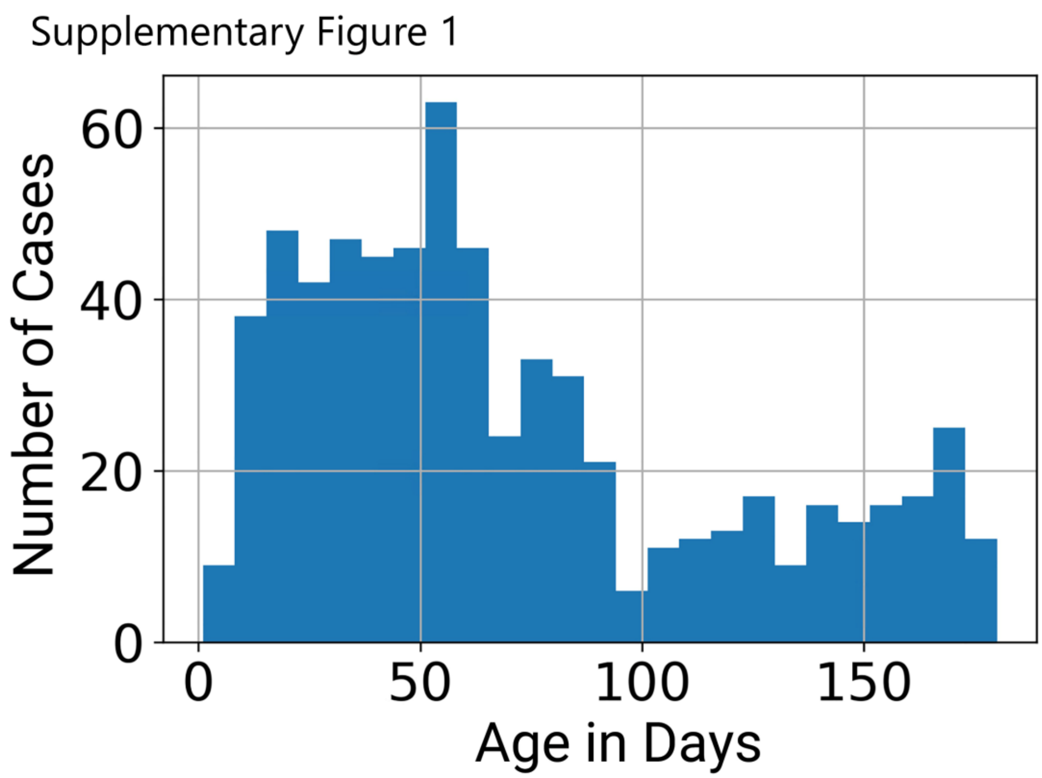

Supplementary Figure 2- Calendarial Distribution of Infant cases and controls matching.

X-axis- calendar date y- axis- in red- the number of cases born on a given day, in green- the number of controls born on a given day, while dark green represents the matched controls (at a ratio of 1:90) Case infants, n=661; Control infants, n=59,460.

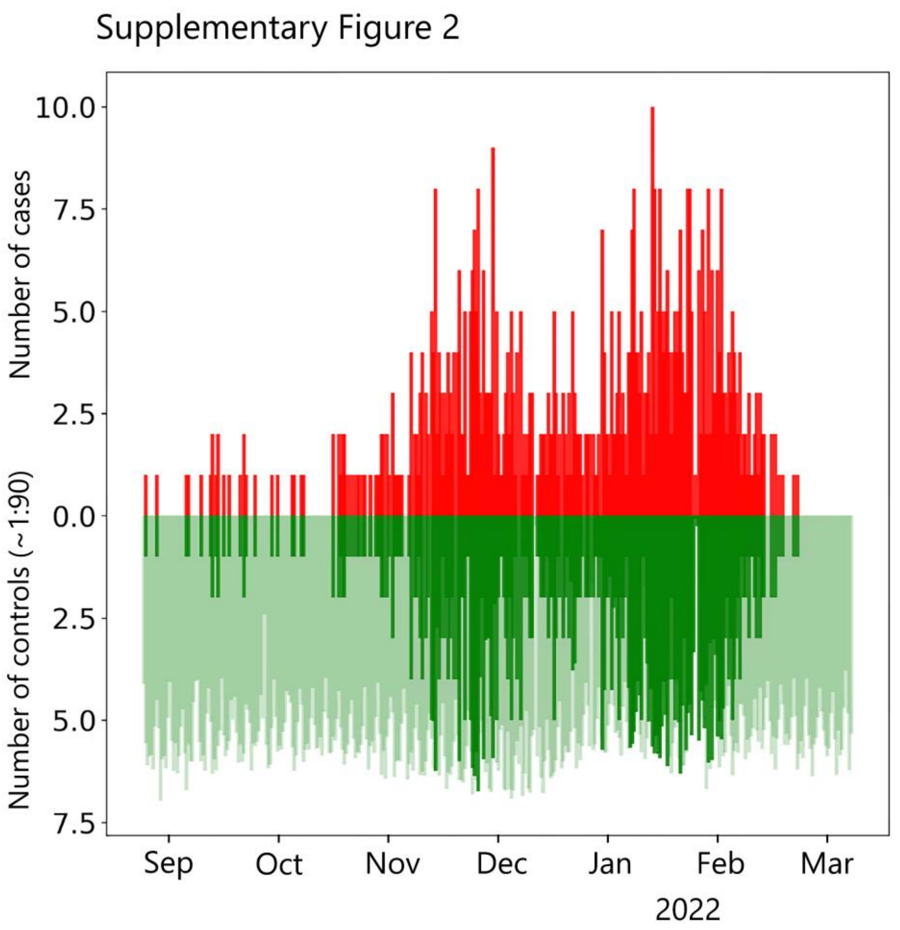

Supplementary Figure 3- A-calendarial distribution of infant population according to maternal immunity group at delivery, in infant cases (A) and controls in a ratio of 1:90 (B). Y-axis represents the number of infants included in the study group by date (x-axis).

C-D: The proportional distribution of the cases and controls into the maternal immunity sub-groups, by date.

E: Stacked bar graph showing the overall proportional distribution into the study immunity sub-groups among cases and controls. (Partial vaccination: 1-2 doses; Full vaccination: 3-4 doses.) Case infants, n=661; Control infants, n=59,460.

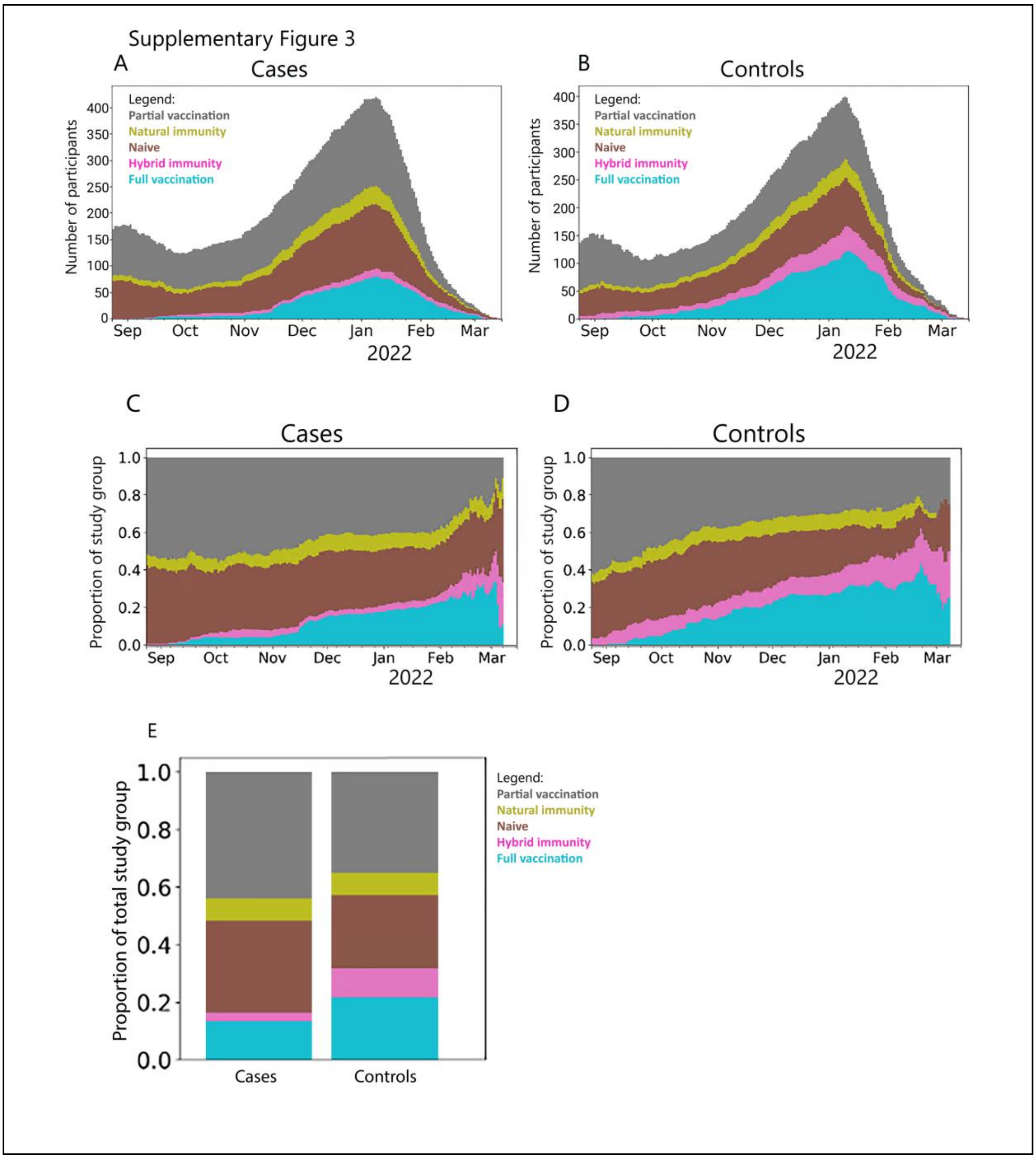

Supplementary Figure 4A-B: Effectiveness of Maternal Natural Immunity and Hybrid immunity Against Infant COVID-19-Related Hospitalization During the First 180 Days of Life, Stratified According to Maternal COVID-19 Infection Timing, during Delta (A) and Omicron (B) waves.

Cox proportional hazards models were used to estimate the hazard ratios (HR) and 95% confidence intervals (CI) for COVID-19 hospitalizations in the Hybrid and Natural groups of maternal immunity status, compared to the maternal naïve group, stratified by maternal infection timing. Infection before pregnancy is defined as the last infection that occurred before conception of pregnancy. Infection before 20 weeks refers to the last documented infection that occurred during the first 20 weeks of gestation, while infection after 20 weeks refers to the last documented infection that occurred during the last 20 weeks of gestation. Models were adjusted for maternal age, gestational age at delivery, parity, and neonatal sex; multifetal gestation, birthweight, and number of maternal documented SARS-CoV-2 tests during pregnancy. Maternal Immunization effectiveness was calculated as (1 − adjusted hazard ratio) x 100. Boxes and error bars represent the median and 95% CI. Dotted line shows no effect point. Subgroups are indicated by color: Hybrid, red; Natural immunity, green; Cases, n=661; Control infants, n=59,460; Delta wave case infants, n=173; Delta wave controls, 15,570; Omicron wave case infants, n=486; Omicron wave controls, n=43,710)

Supplementary Figure 4A: Delta

Subgroup	Case infants<br>Immunity group/ Total cases (%)	Control infants<br>Immunity group/ Total controls (%)	HR (95% CI)	Effectiveness of maternal immunity<br>*% (95% CI)*
Infection ***before*** pregnancy				
**Hybrid**	0/173 (0%)	286/15570 (1.8%)	Ø*	100
**Natural**	1/173 (0.6%)	371/15570 (2.4%)	0.14 (0.02-1.01)	86 (-1 to 98)
Infection ***during*** pregnancy				
**Hybrid**	0/173 (0%)	487/15570 (3.1%)	Ø*	100
**Natural**	6/173 (3.5%)	1096/15570 (7.0%)	0.29 (0.13-0.67)	71 (33 to 87)
Infection ***before 20*** weeks				
**Hybrid**	0/173 (0%)	292/15570 (1.9%)	Ø*	100
**Natural**	5/173 (2.9%)	833/15570 (5.4%)	0.31 (0.13-0.78)	69 (22 to 87)
Infection ***after 20*** weeks				
**Hybrid**	0/173 (0%)	195/15570 (1.3%)	Ø*	100
**Natural**	1/173 0.6%	263/15570 (1.7%)	0.21 (0.03-1.53)	79 (-53 to 97)

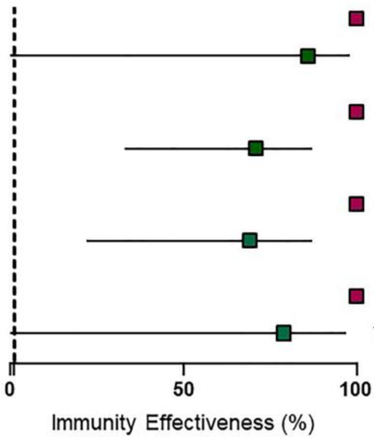

*No cases in Hybrid immunity group

Supplementary Figure 4B: Omicron

Subgroup	Case infants<br>*Immunity group/ total cases (%)*	Control infants<br>*Immunity group/ total control (%)*	HR (95% CI)	Effectiveness of maternal immunity<br>*% (95% CI)*
Infection ***before*** pregnancy				
**Hybrid**	13/486(2.7%)	2639/43710 (6.0%)	0.24 (0.14-0.43	76 (57 to 86)
**Natural**	20/486(4.1%)	2132/43710 (4.9%)	0.48 (0.3-0.78)	52 (22 to 70)
Infection ***during*** pregnancy				
**Hybrid**	7/486 (1.4%)	2386/43710 (5.5%)	0.13 (0.06-0.28	87 (72 to 94)
**Natural**	19/486 (3.9%)	1799/43710 (4.1%)	0.57 (0.35-0.93)	43 (7 to 65)
Infection ***before 20*** weeks				
**Hybrid**	3/486 (0.6%)	592/43710 (1.4%)	0.24 (0.08-0.77	76 (23 to 92)
**Natural**	11/486 (2.3%)	815/43710 (1.9%)	0.82 (0.44-1.53)	18 (-53 to 56)
Infection ***after 20*** weeks				
**Hybrid**	4/486 (0.8%)	1794/43710 (4.1%)	0.1 (0.04-0.26)	90 (74 to 96)
**Natural**	8/486 (1.6%)	984/43710 (2.3%)	0.4 (0.19-0.82)	60 (18 to 81)

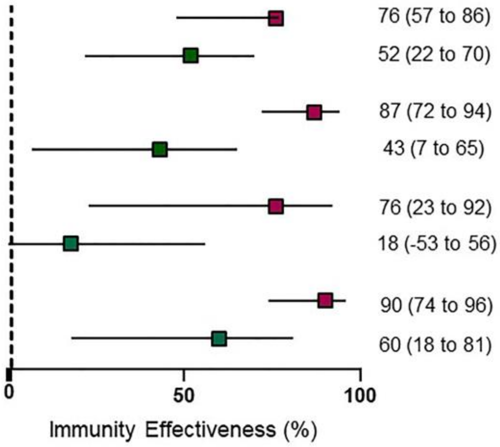

Supplementary Figure 5: Effectiveness of Maternal Hybrid Immunity (i.e., COVID-19 vaccination and infection) Against Infant COVID-19-Related Hospitalization During the First 180 Days of Life, Stratified According to Maternal COVID-19 Immune Sequence or Last Stimulation.

Cox proportional hazards models were used to estimate the hazard ratios (HR) and 95% confidence intervals (CI) for COVID-19 hospitalizations in the Hybrid maternal immunity status, compared to the maternal naïve group, stratified by maternal immunity sequence or last stimulation. Infection or vaccination before, refers to the first immune stimulation; Last stimulation before 20 weeks refers to the last documented stimulation that occurred during the first 20 weeks of gestation, while last stimulation after 20 weeks refers to the last documented stimulation that occurred during the last 20 weeks of gestation. Models were adjusted for maternal age, gestational age at delivery, parity, and neonatal sex; multifetal gestation, birthweight, and number of maternal documented SARS-CoV-2 tests during pregnancy. Maternal Immunization effectiveness was calculated as (1 − adjusted hazard ratio) x 100. Boxes and error bars represent the median and 95% CI. Dotted line shows no effect point. Subgroup comparisons are indicated by color. (Case infants, n=661; Control infants, n=59,460.)

Supplementary Figure 5

Subgroup	Case infants *Immunity group/ total cases (%)*	Control infants *Immunity group/ total control (%)*	HR (95% CI)	Effectiveness of maternal immunity *% (95% CI)*
**Hybrid**	20/661 (3.0%)	5832/59460 (9.8%)	0.16 (0.10-0.25)	84 (75 to 90)
Infection *before* vaccine	13/661 (2.0%)	3270/59460 (5.5%)	0.20 (0.11-0.35)	80 (65 to 89)
Vaccine *before* infection	7/661 (1.1%)	2562/59460 (4.3%)	0.12 (0.05-0.25)	88 (75 to 95)
Last stimulation- ***before pregnancy***	2/661 (0.3%)	340/59460 (0.6%)	0.26 (0.06-1.06)	74 (-6 to 94)
Last stimulation- ***during pregnancy***	18/661 (2.7%)	5492/59460 (9.2%)	0.16 (0.10-0.26)	84 (74 to 90)
Last stimulation- ***before 20 weeks***	6/661 (0.9%)	1347/59460 (2.3%)	0.22 (0.10-0.49)	78 (51 to 90)
Last stimulation- ***after 20 weeks***	12/661 (1.8%)	4145/59460 (7.0%)	0.14 (0.08-0.26)	86 (74 to 92)
Last vaccination- ***before 20 weeks***	4/661 (0.6%)	945/59460 (1.6%)	0.21 (0.08-0.58)	79 (42 to 92)
Last vaccination- ***after 20 weeks***	8/661 (1.2%)	2098/59460 (3.5%)	0.20 (0.10-0.41)	80 (59 to 90)

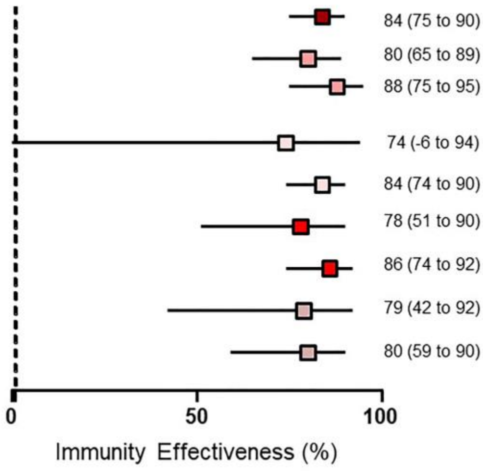

Supplement: Supplementary file 1 — Supplementary Information [file 41467_2024_46694_MOESM1_ESM.pdf]
