# Supplementary material for: Maternal hybrid immunity and risk of infant COVID-19 hospitalizations: national case-control study in Israel
Source: Nat Commun. 2024 Apr 2;15:2846. doi: 10.1038/s41467-024-46694-x (PMC10987618; doi:10.1038/s41467-024-46694-x)
Supplement: Supplementary file 3 — Reporting Summary [file 41467_2024_46694_MOESM3_ESM.pdf]

nature portfolio

Corresponding author(s): Prof. Michal Lipschuetz, Prof. Ofer Beharier

Last updated by author(s): 25 Feb. 2024

Reporting Summary

Nature Portfolio wishes to improve the reproducibility of the work that we publish. This form provides structure for consistency and transparency in reporting. For further information on Nature Portfolio policies, see our Editorial Policies and the Editorial Policy Checklist.

Statistics

For all statistical analyses, confirm that the following items are present in the figure legend, table legend, main text, or Methods section.

n/a	Confirmed	
☐	☒	The exact sample size (*n*) for each experimental group/condition, given as a discrete number and unit of measurement
☐	☒	A statement on whether measurements were taken from distinct samples or whether the same sample was measured repeatedly
☐	☒	The statistical test(s) used AND whether they are one- or two-sided<br>*Only common tests should be described solely by name; describe more complex techniques in the Methods section.*
☐	☒	A description of all covariates tested
☒	☐	A description of any assumptions or corrections, such as tests of normality and adjustment for multiple comparisons
☐	☒	A full description of the statistical parameters including central tendency (e.g. means) or other basic estimates (e.g. regression coefficient) AND variation (e.g. standard deviation) or associated estimates of uncertainty (e.g. confidence intervals)
☐	☒	For null hypothesis testing, the test statistic (e.g. *F*, *t*, *r*) with confidence intervals, effect sizes, degrees of freedom and *P* value noted<br>*Give P values as exact values whenever suitable.*
☒	☐	For Bayesian analysis, information on the choice of priors and Markov chain Monte Carlo settings
☒	☐	For hierarchical and complex designs, identification of the appropriate level for tests and full reporting of outcomes
☒	☐	Estimates of effect sizes (e.g. Cohen's *d*, Pearson's *r*), indicating how they were calculated

Our web collection on statistics for biologists contains articles on many of the points above.

Software and code

Policy information about availability of computer code

Data collection	No software was used for data collection
Data analysis	Python version 3.7.3 and lifelines 0.24.14 and IBM-SPSS for Windows, version 29 (IBM Corp., Armonk, N.Y., USA)

For manuscripts utilizing custom algorithms or software that are central to the research but not yet described in published literature, software must be made available to editors and reviewers. We strongly encourage code deposition in a community repository (e.g. GitHub). See the Nature Portfolio guidelines for submitting code & software for further information.

Data

Policy information about availability of data

All manuscripts must include a data availability statement. This statement should provide the following information, where applicable:

- Accession codes, unique identifiers, or web links for publicly available datasets
- A description of any restrictions on data availability
- For clinical datasets or third party data, please ensure that the statement adheres to our policy

Due to national and organizational data privacy regulations, individual-level data such as those used for this study cannot be shared openly.

Research involving human participants, their data, or biological material

Policy information about studies with human participants or human data. See also policy information about sex, gender (identity/presentation), and sexual orientation and race, ethnicity and racism.

Reporting on sex and gender	This study exclusively focuses on the vaccination of parturients and their infants' COVID infection, with neonatal assigned sex at birth being accounted for in Table 1.
Reporting on race, ethnicity, or other socially relevant groupings	The study is based on a national database covering all citizens, without exclusions based on any social/demographic grouping.
Population characteristics	We conducted a national, population-based, case-control study with basic characteristics as age, parity, gestational age, vaccination and COVID infection status. Study included infants up to the age of 180 days.
Recruitment	Participants were not recruited, but rather selected from a retrospective national database. All eligible participants were considered. As with any observational study, there exists the potential for residual bias, even after adjustment for potential confounders.
Ethics oversight	This study was approved by the Hadassah Medical Center institutional review board and it was deemed exempt from the requirement for informed consent.

Note that full information on the approval of the study protocol must also be provided in the manuscript.

Field-specific reporting

Please select the one below that is the best fit for your research. If you are not sure, read the appropriate sections before making your selection.

☐ Life sciences ☒ Behavioural & social sciences ☐ Ecological, evolutionary & environmental sciences

For a reference copy of the document with all sections, see nature.com/documents/nr-reporting-summary-flat.pdf

Behavioural & social sciences study design

All studies must disclose on these points even when the disclosure is negative.

Study description	This is a retrospective cohort analysis, based on existing medical records. Methods are strictly quantitative.
Research sample	This is a nationwide population-based case-control study. The study includes all infants hospitalized with COVID-19 during the study period (up to age 180 days) and controls. Sample is based on all citizens without other exclusions for any social / demographic parameters. The database is compiled by the Israel Ministry of Health.
Sampling strategy	Case infants were defined as those with documented hospitalization due to COVID-19. For every case infant, approximately 90 infants were individually matched, based on birthdate (+/-3 days). Control infants were randomly selected from the birthdate appropriate population of infants who were not hospitalized for COVID-19. We present the full set of characteristics of the population stratified by exposure group in Table 1. All cases recorded in the national registry that complied with inclusion criteria (i.e. age up to 180 days, GA>23 weeks, birthweight>500 g, documented birthweight) were included.
Data collection	All data used in this study is based on existing national databases. No dedicated data collection was performed. Data was extracted according to the inclusion and exclusion criteria defined. Given the retrospective nature of the study, no blinding was performed.
Timing	We included infants younger than six months of age who were born between February 27, 2021, i.e. six months prior to August 24, 2021, and March 15, 2022 (See Flow Chart, extended data- Figure 1). There was no gap in collection of cases.
Data exclusions	We excluded infants who were born before 23 weeks of gestation, had a birthweight of less than 500 grams, missing birthweight, or were hospitalized due to COVID-19 prior to August 24, 2021.
Non-participation	None of the 60,121 infants who met the eligibility criteria were excluded from the analysis.
Randomization	This is an observational study, no randomization was performed because all eligible cases were included.

Reporting for specific materials, systems and methods

We require information from authors about some types of materials, experimental systems and methods used in many studies. Here, indicate whether each material, system or method listed is relevant to your study. If you are not sure if a list item applies to your research, read the appropriate section before selecting a response.

Materials & experimental systems

n/a	Involved in the study
☒	☐ Antibodies
☒	☐ Eukaryotic cell lines
☒	☐ Palaeontology and archaeology
☒	☐ Animals and other organisms
☒	☐ Clinical data
☒	☐ Dual use research of concern
☒	☐ Plants

Methods

n/a	Involved in the study
☒	☐ ChIP-seq
☒	☐ Flow cytometry
☒	☐ MRI-based neuroimaging

Plants

Seed stocks	N/A`
Novel plant genotypes	N/A
Authentication	N /A
